# Relationship among LRP1 expression, Pyk2 phosphorylation and MMP‐9 activation in left ventricular remodelling after myocardial infarction

**DOI:** 10.1111/jcmm.13113

**Published:** 2017-04-04

**Authors:** Elena Revuelta‐López, Carol Soler‐Botija, Laura Nasarre, Aleyda Benitez‐Amaro, David de Gonzalo‐Calvo, Antoni Bayes‐Genis, Vicenta Llorente‐Cortés

**Affiliations:** ^1^ Cardiovascular Research Center CSIC‐ICCC IIB Sant Pau Hospital de la Santa Creu i Sant Pau Barcelona Spain; ^2^ ICREC (Heart Failure and Cardiac Regeneration) Research Program Health Sciences Research Institute Germans Tries i Pujol Badalona (Barcelona) Spain; ^3^ Cardiology Service Germans Trias i Pujol University Hospital Badalona Spain; ^4^ Department of Medicine Universitat Autònoma de Barcelona Barcelona Spain

**Keywords:** LRP1, MMP‐9, cardiac remodelling, myocardial infarction, pPyk2

## Abstract

Left ventricular (LV) remodelling after myocardial infarction (MI) is a crucial determinant of the clinical course of heart failure. Matrix metalloproteinase (MMP) activation is strongly associated with LV remodelling after MI. Elucidation of plasma membrane receptors related to the activation of specific MMPs is fundamental for treating adverse cardiac remodelling after MI. The aim of current investigation was to explore the potential association between the low‐density lipoprotein receptor‐related protein 1 (LRP1) and MMP‐9 and MMP‐2 spatiotemporal expression after MI. Real‐time PCR and Western blot analyses showed that LRP1 mRNA and protein expression levels, respectively, were significantly increased in peri‐infarct and infarct zones at 10 and 21 days after MI. Confocal microscopy demonstrated high colocalization between LRP1 and the fibroblast marker vimentin, indicating that LRP1 is mostly expressed by cardiac fibroblasts in peri‐infarct and infarct areas. LRP1 also colocalized with proline‐rich tyrosine kinase 2 (pPyk2) and MMP‐9 in cardiac fibroblasts in ischaemic areas at 10 and 21 days after MI. Cell culture experiments revealed that hypoxia increases LRP1, pPyk2 protein levels and MMP‐9 activity in fibroblasts, without significant changes in MMP‐2 activity. MMP‐9 activation by hypoxia requires LRP1 and Pyk2 phosphorylation in fibroblasts. Collectively, our *in vivo* and *in vitro* data support a major role of cardiac fibroblast LRP1 levels on MMP‐9 up‐regulation associated with ventricular remodelling after MI.

## Introduction

Adverse myocardial remodelling is associated with poor patient outcomes in the setting of ischaemic heart disease and/or MI, cardiac hypertrophy and cardiomyopathy disease processes 1, 2, 3. In particular, adverse cardiac remodelling after MI causes ventricular functional impairment and heart failure (HF). The incidence of HF after MI is determined by infarct area size, infarct wound healing and chronic LV remodelling 4, 5, 6.

Cardiomyocyte apoptosis and necrosis involving infarcted regions and residual viable myocardium trigger a cascade of immune‐inflammatory pathways and cellular mechanisms that promote wound healing and a unique pattern of LV structural remodelling‐related changes 7, 8, 9. During its early phases, LV remodelling occurs secondary to thinning and dilation of the infarcted myocardial wall (infarct expansion). During its late phases, LV remodelling occurs secondary to architectural rearrangements of the surviving myocardium characterized by myocyte hypertrophy, interstitial fibrosis and LV dilation 4, 5. Perivascular fibrosis impairs myocyte oxygen availability, reduces coronary reserve and exacerbates myocardial ischaemia 10. LV remodelling thus plays a central role in clinical progression to HF. The identification of new receptors involved in LV remodelling has opened the door for finding new therapeutic targets for treating HF. Low‐density LRP1 is a lipoprotein receptor up‐regulated by hypoxia in human vascular smooth muscle cells (hVSMCs) 11, 12 and cardiomyocytes 13, 14. LRP1 was strongly up‐regulated in ischaemic myocardial tissue in a porcine model of acute MI 13, 14 and in ischaemic cardiomyopathy patients 13, 14. LRP1 plays a crucial role in regulating MMP‐9 and MMP‐2 expression 15, 16, 17 and mediates MMP‐9 cellular catabolism to regulate its extracellular levels 18, 19. Our group recently demonstrated that LRP1 modulates MMP‐9 expression and activation in hVSMCs exposed to hypoxia through Pyk2 phosphorylation 12. It is known that MMP‐9 plays a key role in MI‐related cardiac remodelling and adverse outcomes 20, 21; however, neither LRP1 spatiotemporal expression nor the relationship between LRP1 and MMP‐9 expression during the evolution of adverse myocardial remodelling has been previously investigated. The aim of current investigation was to explore the potential relationship between cardiac LRP1 spatiotemporal expression after MI and MMP‐9 and MMP‐2 expression and activation.

## Materials and methods

### Mouse model of MI

A total of 46 male C57/Bl6 mice 12–13 weeks of age (25–30 g; Charles River Laboratories, Inc.; Wilmington, MA) were used in this study. MI was induced as previously described 22. Briefly, each animal was intubated and anesthetized with a mixture of O_2_/isoflurane and was mechanically ventilated. The heart was exposed, and the left anterior descending coronary artery was permanently occluded. The animals were sacrificed at 1 day (*n* = 17), 10 days (*n* = 14) and 21 days (*n* = 15) after operation, and their hearts were excised and frozen in liquid nitrogen for molecular and lipid analyses. Six animals from each group were processed for histological analysis; their hearts were arrested during diastole using cardioplegic solution (68.4 mM NaCl, 59 mM KCl, 11.1 mM glucose, 1.9 mM NaHCO_3_, 29.7 mM 2,3‐butanedione monoxime, 1000 U heparin), excised, fixed, cryopreserved in 30% sucrose in phosphate‐buffered saline, embedded in Tissue‐Tek O.C.T. (Sakura) and snap‐frozen in liquid nitrogen‐cooled isopentane for histological evaluation. All animal handling procedures were approved by the Institutional Animal Care and Use Committee and conformed to the Guide for the Care and Use of Laboratory Animals of the Institute of Laboratory Animal Research (NIH Pub. No. 86‐23, Revised 1996).

### Macrophage cell culture

Monocyte‐derived macrophages were isolated by standard protocols from buffy coats (35–40 ml) of healthy donors. This protocol complied with the Declaration of Helsinki and was approved by the local institutional committee on human research. Cells were applied on 15 ml of Ficoll–Hypaque and centrifuged at 400×*g* for 40 min. at 22°C, with no brake. Mononuclear cells were obtained from the central white band of the gradient, exhaustively washed in PBS and resuspended in RPMI medium supplemented with 10% human AB serum, 1% P/S and 1% HEPES. Cells were allowed to differentiate into macrophages by exposure to 10% human AB serum for 7 days, changing the medium every other day.

### Fibroblast cell culture

Control (*Lrp1* ^+/+^, MEF, CRL‐2214) and LRP1‐deficient fibroblasts (*Lrp1* ^−/−^, PEA13, CRL‐2216) fibroblasts were obtained from American Type Culture Collection. *Lrp1* ^+/+^ and *Lrp1* ^−/−^ were grown in DMEM supplemented with 10% foetal calf serum, 2 mM L‐glutamine, 100 U/ml penicillin G and 100 μg/ml streptomycin, as previously described 23. The cells were exposed to hypoxia (1% O_2_) in an H35 Hypoxic/Anoxic Workstation (Don Whitley Scientific Ltd.; West Yorkshire, UK) with 94% N_2_ and 5% CO_2_. The cells were then harvested *via* scraping in TriPure™ Isolation Reagent (Roche Molecular Diagnostics; Indianapolis, USA) for PCR and Western blot analysis, and the cell culture supernatants were collected for zymography studies.

### Cell exposure to normoxic and hypoxic conditions

Macrophages and fibroblasts (*Lrp1* ^+/+^ and *Lrp1* ^−/−^) were serum‐deprived once they reached 80% confluence. Cells were exposed to normoxia (21% O_2_) in an incubator with gas mixtures consisting of 74% N_2_ and 5% CO_2_ or to hypoxia (1% O_2_) in a Hypoxic/Anoxic Workstation: H35 (Don Whitley Scientific Ltd.) with 94% N_2_ and 5% CO_2_. Cells were then harvested by scraping in TriPure™ Isolation Reagent (Roche Molecular Diagnostics) for Western blot analysis. Culture supernatants were finally collected for zymographic studies.

### RNA extraction and cDNA synthesis

Frozen cardiac tissue samples were pulverized using a mortar and a pestle in liquid nitrogen to maintain RNA integrity. Cardiac tissue (40 mg) was weighed, and total RNA was isolated using TriPure™ Isolation Reagent (Roche Molecular Diagnostics, Indianapolis, USA), according to the manufacturer's instructions. RNA yield and quality were assessed by 1% agarose gel electrophoresis, and then the RNA was stored at −80°C until analysis. Reverse transcription was performed using 1.5 μg of total RNA and a High‐capacity cDNA Reverse Transcription Kit (Applied Biosystems, Foster City, CA, USA). The cDNA was stored at −20°C.

### Gene expression analyses by RT‐PCR

*Lrp1, Mmp‐9* and *Mmp‐2* mRNA gene expression analyses were performed *via* semiquantitative real‐time reverse‐transcriptase polymerase chain reaction (RT‐PCR) using the assays‐on‐demand listed in Table 1. Endogenous eukaryotic 18S rRNA expression served as an internal gene amplification control. RT‐PCR was performed using 1 μl of reverse transcription products mixed with 10 μl of TaqMan Universal PCR Master Mix (Applied Biosystems, Foster City, CA, USA), 1 μl of 20× assays and 8 μl of nuclease‐free water. After gentle mixing, the mixture was transferred to a real‐time PCR microplate.

**Table 1:** Assays‐on‐demand used for real‐time PCR analysis

Assays	Reference	Species	Company
*Lrp1*	Mm00464608_m1	Mouse	Applied Biosystems
*Mmp‐9*	Rn00675898_m1	Rat	Applied Biosystems
*Mmp‐2*	Rn01538169_m1	Rat	Applied Biosystems
18s rRNA	4319413E		Applied Biosystems

PCR was performed in a PCR‐7600HT sequence detection system (Abiprism; Applied Biosystems) under the following conditions: 50°C for 2 min., 95°C for 10 min., 40 cycles at 95°C for 15 sec. and 60°C for 1 min. Relative gene expression levels were quantified and analysed using SDS 2.4 software, and the real‐time values of each sample were averaged and compared using the C_T_ method, by which the target RNA expression level (2^−ΔΔCT^) was normalized to that of an endogenous control (ΔCT).

### Western blotting

Frozen cardiac tissue samples were pulverized using a mortar and a pestle in liquid nitrogen to maintain protein integrity. Pulverized tissue aliquots (40 mg) were subsequently weighed, and protein was isolated using TriPure™ Isolation Reagent (Roche Molecular Diagnostics), according to the manufacturer's instructions. The protein was quantified using Pierce BCA Protein Assay (Thermo Scientific, Waltham, MA, USA). Equivalent amounts of total protein (25 or 30 μg) were loaded onto 10% (v/v) SDS‐polyacrylamide gels under reducing conditions. The samples were then electrotransferred to nitrocellulose membranes, which were saturated at room temperature for 1 hr in TTBS (20 mM Tris–HCl, pH 7.5, 500 mM NaCl, 0.01% Tween 20 and 5% non‐fat milk). Western blot analyses were performed using specific monoclonal antibodies (Table 2) and the corresponding secondary antibodies (1:10,000 dilution; Dako; Glostrup, Denmark).

**Table 2:** Primary antibodies used for Western blot analysis

Antibody	References	Company
LRP1	Ab92544	Abcam
pPyk2	#3291	Cell Signaling Technology
Total Pyk2	#3480	Cell Signaling Technology
pERK1,2	#9101	Cell Signaling Technology
Total ERK1,2	KAP‐MA001	Assay Design
Troponin T	#MS‐295‐P	Thermo Scientific

Equal protein loading was verified *via* Ponceau staining and Western blotting for troponin T. Bands were detected using ECL Prime Western Blotting Detection Reagent (Amersham) and quantified *via* densitometry using a ChemiDoc system and Quantity One software (Bio‐Rad, Hercules, CA, USA). The results are expressed as arbitrary units of intensity.

### Gelatin zymography

Relative MMP‐9 and MMP‐2 activity levels in infarcted myocardium were measured by zymography. Pulverized tissue aliquots (5 mg) were weighed, homogenized in 60 μl of lysis buffer [1 M Tris–HCl, pH 8, 1 M KCl supplemented with one tablet of complete protease inhibitor cocktail (Roche Molecular Diagnostics, IN, USA)], sonicated and centrifuged at 16, 000 × g for 15 min. The supernatants were subsequently quantified *via* Pierce BCA Protein Assay (Thermo Scientific, Waltham, MA, USA). Thirty micrograms of protein was then mixed with 6× non‐reducing loading buffer, after which the samples were loaded onto 10% (v/v) SDS‐polyacrylamide gels, with 1 mg/ml porcine skin A gelatin (Sigma‐Aldrich , St. Luis, MO, USA) serving as a substrate for MMP enzymatic activity, and run at 4°C for 4–6 hrs.

After electrophoresis, the gels were rinsed twice in 2.5% Triton X‐100 for 30 min. at room temperature and then incubated in substrate buffer (50 mM Tris–HCl, 10 mM CaCl_2_, 0.02% (w/v) N_3_Na, pH 8) for 18–20 hrs at 37°C. The gels were dyed with 10% acetic acid with one tablet of PhastGel™ Blue R. Gelatinolytic activity areas, in which the protease digested the substrate, appeared as clear bands on a blue background. The gels were ultimately scanned with a GS‐800 calibrated imaging densitometer, and quantitative densitometric analysis of the digested bands was performed using Quantity‐One software (Bio‐Rad, Hercules, CA, USA).

### Immunofluorescence

Mouse heart cryosections were incubated with primary antibodies against LRP1 (2 μg/ml; Abcam, Cambridge, UK), cardiac troponin I (cTnI; 2 μg/ml; Abcam), vimentin (2 μg/ml; Abcam, Cambrigde, UK), pPky2 (4 μg/ml; Santa Cruz Biotechnology, Dallas, TX, USA), pERK1,2 (8 μg/ml; Santa Cruz Biotechnology, Dallas, TX, USA), MMP‐9 (20 μg/ml; AMS Biotechnology , Abingdon, UK) and MMP‐2 (20 μg/ml; AMS Biotechnology , Abingdon, UK) (Table 3). These samples were also stained with biotinylated GSLI B4 isolectin (Vectors Laboratories) for vessel detection. Cell nuclei were counterstained with 4′,6‐diamidino‐2‐phenylindole (DAPI), and the results were analysed with an Axio Observer Z1 (Zeiss) laser confocal microscope.

**Table 3:** Primary antibodies used for immunohistochemical analysis

Antibody	References	Company
LRP1	ab92544	Abcam
LRP1	10R‐L107a	Fitzgerald
Vimentin	ab89996	Abcam
pPyk2	sc‐16824	Santa Cruz Biotechnology
MMP‐9	TP221	AMS Biotechnology
pERK1,2	sc‐16982	Santa Cruz Biotechnology
MMP‐2	TP220	AMS Biotechnology
cTroponin I	Ab188877	Abcam

### Statistical analysis

The statistical software package SPSS 15.0 for Windows (SPSS Inc., Chicago, IL, USA) was used for all statistical analyses. Data normality was analysed using the Kolmogorov–Smirnov test. Continuous variables were compared between groups using one‐way anova. Comparisons between each subgroup were performed using Tukey *post hoc* test, and independent samples were analysed using Student's *t*‐test. Data are presented as the mean ± S.D. *P* < 0.05 was considered statistically significant.

## Results

### *Lrp1* expression is up‐regulated in ischaemic myocardium at 10 and 21 days after MI

**Figure 1:**
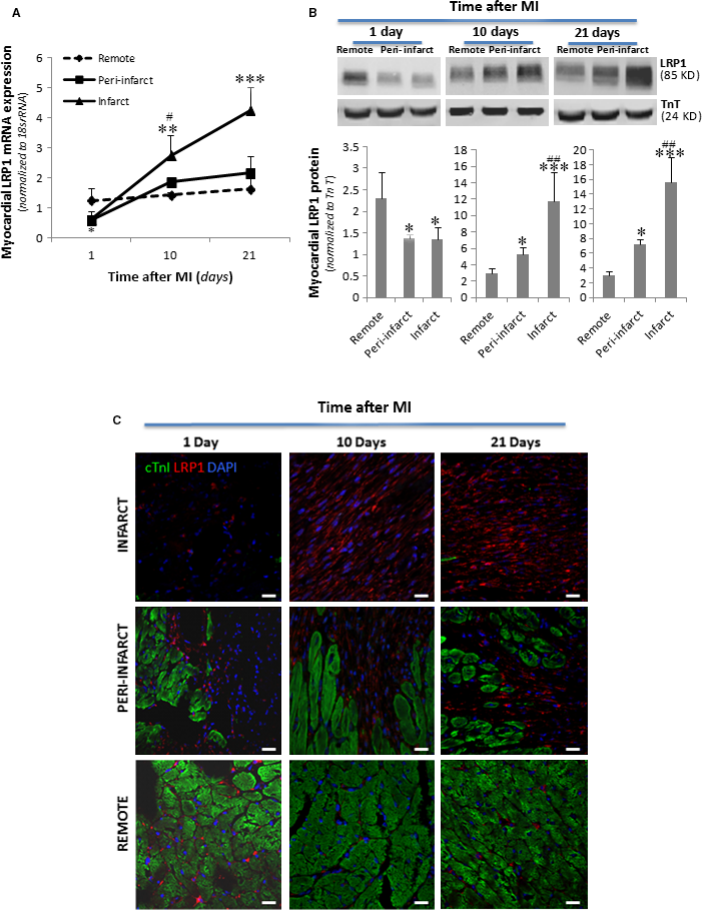
Changes in *Lrp1* mRNA and LRP1 protein levels after MI. Frozen myocardial tissue samples (≈ 40 mg) from the remote, peri‐infarct and infarct zones were homogenized in TriPure™ Reagent. RNA and protein were isolated as explained in the Materials and Methods. (**A**) RT‐PCR showing *Lrp1* mRNA expression levels. Data were processed using a specially designed software program based on the Ct values of each sample and normalized to 18s rRNA, which served as an endogenous control. (**B**) Representative Western blot analysis results showing LRP1 and troponin T protein expression and bar graphs showing the mean ± S.D. of LRP1 protein bands normalized to troponin T bands. *n* = 8. *P < 0.05 *versus* remote; ^#^ *P* < 0.05 *versus* peri‐infarct; **P < 0.01 *versus* remote; ^##^ *P* < 0.01 *versus* peri‐infarct; ***P < 0.005 *versus* remote. (**C**) Representative immunostaining of heart cross sections showing the temporal evolution of LRP1 levels after MI. LRP1 is shown in red, and cTnI, in green. Cell nuclei were counterstained with DAPI (blue). Scale bars, 20 μm.

**Figure 2:**
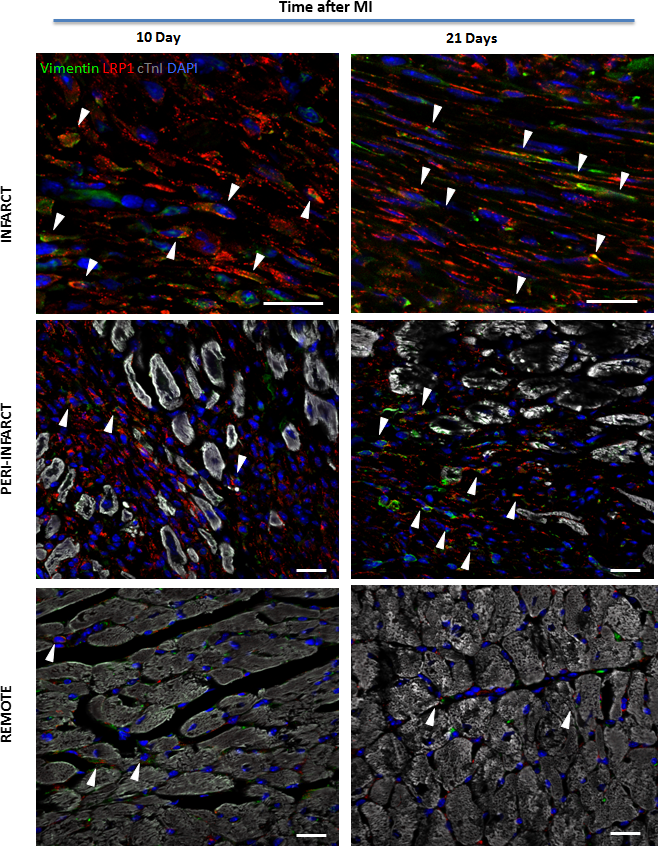
Identification of LRP1‐expressing cells in infarcted heart cross sections. LRP1 is shown in red; vimentin, in green; cTnI, in grey; and Lrp1 and vimentin colocalization, in yellow. Cell nuclei were counterstained with DAPI (blue). Scale bars, 20 μm.

*Lrp*1 expression levels were analysed by RT‐PCR, and LRP1 protein expression levels in the non‐infarct or remote peri‐infarct and infarct regions were determined by Western blotting and confocal laser microscopy in all experimental groups (1, 10 and 21 days after MI). These analyses demonstrated significant increases in *Lrp1* gene expression (Fig. 1A) and LRP1 protein levels (Fig. 1B) in the peri‐infarct and infarct regions compared to the non‐infarct remote zones at 10 and 21 days after MI. These results were confirmed by immunofluorescence. Confocal microscopy images demonstrated dramatic increases in LRP1 levels in the cells forming the scar at 10 and 21 days after MI (Fig. 1C). Slight LRP1 expression was also observed in the fibroblasts surrounding the remote cardiomyocytes. Precise colocalization of the fibroblast marker vimentin and LRP1 demonstrated that LRP1 is mostly expressed in cardiac fibroblasts (Fig. 2).

RT‐PCR and Western blot analysis showed that LRP1 mRNA and protein expression levels, respectively, were significantly reduced in the infarct zone at 1 day after MI, in contrast to the above results (Fig. 1A and B). Immunofluorescence imaging confirmed the reductions in LRP1 protein levels in the infarct areas compared to the peri‐infarct or remote areas (Fig. 1C).

### pPyk2 and pERK1,2 are signal mediators that are differentially modulated at both the temporal level and the spatial level after MI

**Figure 3:**
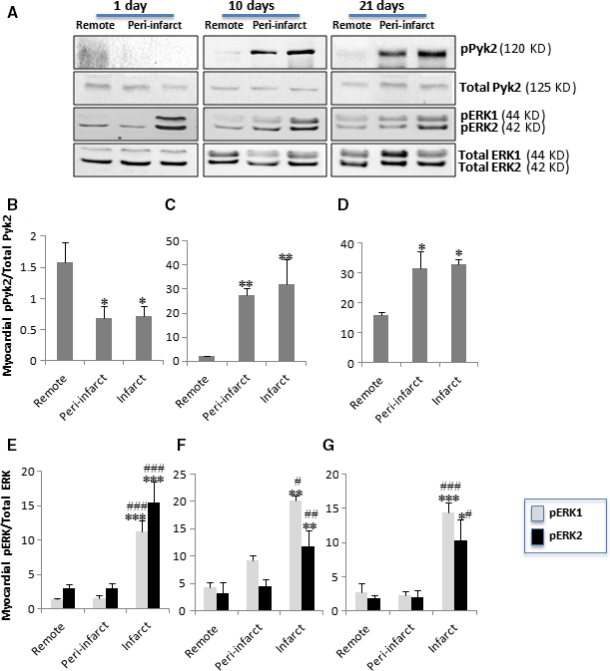
Temporal and spatial evolution of myocardial phosphorylated Pyk2 and ERK1,2 levels after MI. (**A**) Representative Western blot analysis showing pPyk2, total Pyk2, pERK1,2 and total ERK1,2 protein expression. Bar graphs showing the mean ± S.D. of the pPyk2/total Pyk2 ratio and the pERK1,2/total ERK1,2 ratio in the remote, peri‐infarct and infarct zones at 1 (**B, E**), 10 (**C, F**) and 21 (**D, G**) days after MI. *n* = 8. *P < 0.05 *versus* remote; ^#^ *P* < 0.05 *versus* peri‐infarct; **P < 0.01 *versus* remote; ^##^ *P* < 0.01 *versus* peri‐infarct; ***P < 0.005 *versus* remote; ^###^ *P* < 0.005 *versus* peri‐infarct.

Western blot analysis showed that both, LRP1 and pPyk2 levels were significantly down‐regulated in the ischaemic zones at 1 day after MI (Fig. 3A and B). In contrast, pPyk2 expression was strongly up‐regulated in the peri‐infarct and infarct areas at 10 days (Fig. 3A and C) and 21 days after MI (Fig. 3A and D). In addition, pERK1,2 expression was strongly up‐regulated in the infarct areas at 1 (Fig. 3A and E), 10 (Fig. 3A and F) and 21 (Fig. 3A and G) days after MI.

### MMP‐9 and MMP‐2 have a differential temporal and spatial modulation after MI

**Figure 4:**
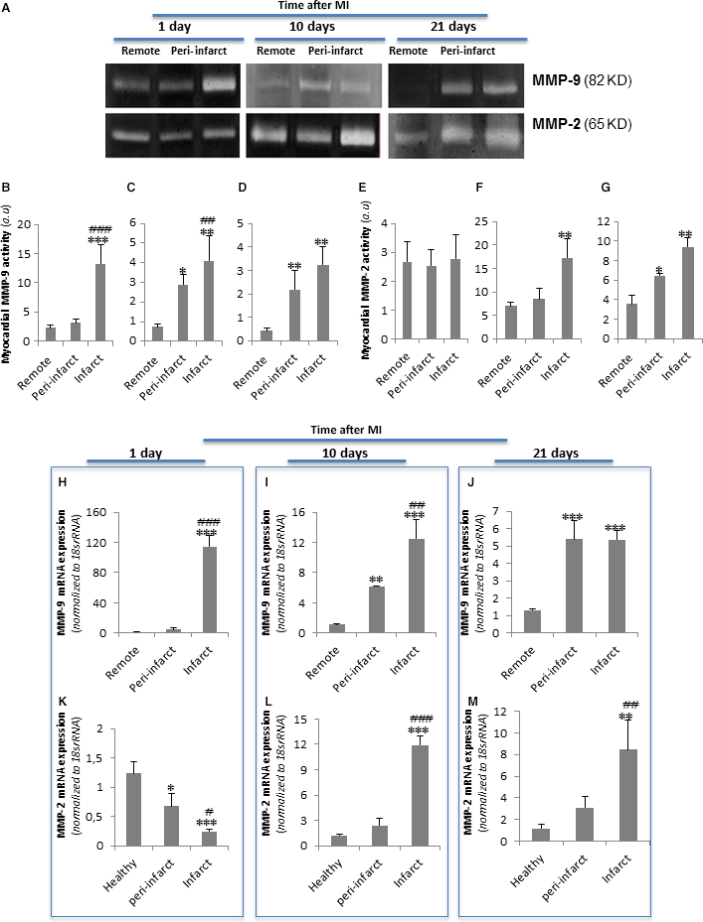
Temporal and spatial evolution of myocardial MMP‐9 and MMP‐2 activation and expression after MI. (**A**) Representative zymography analysis results showing myocardial MMP‐9 and MMP‐2 activity. Bar graphs showing the mean ± S.D. of MMP‐9 and MMP‐2 activity in the remote, peri‐infarct and infarct zones at 1 (**B** and **E**), 10 (**C** and **F**) and 21 (**D** and **G**) days after MI. (**H–M**) Real‐time PCR showing *MMP‐9* and *MMP‐2* mRNA expression levels. Data were processed using a specially designed software program based on the Ct values of each sample and normalized to 18s rRNA, which served as an endogenous control. The results are shown as the mean ± S.D. of *MMP‐9* and *MMP‐2* mRNA expression levels in the remote, peri‐infarct and infarct zones at 1 (**H** and **K**), 10 (**I** and **L**) and 21 (**J** and **M**) days after MI. *n* = 8. *P < 0.05 *versus* remote; **P < 0.01 *versus* remote; ^##^ *P* < 0.01 *versus* peri‐infarct; ***P < 0.005 *versus* remote; ^###^ *P* < 0.005 *versus* peri‐infarct.

Zymography analysis showed that MMP‐9 activity was strongly increased in the infarct areas on day 1 after MI (Fig. 4A and B) and was moderately increased in the peri‐infarct and infarct areas on day 10 (Fig. 4A and C) and day 21 after MI (Fig. 4A and D) compared to the remote zone. Differently, MMP‐2 activity was extremely low in all tested zones on day 1 after MI (Fig. 4A and E). A strong increase in MMP‐2 activity was detected in all zones at 10 days after MI but especially in the infarct area (Fig. 4A and F). MMP‐2 overactivation was slightly maintained at 21 days after MI (Fig. 4A and G). RT‐PCR analysis yielded similar results than zymography with respect to temporal and spatial MMP‐9 (Fig. 6H, I and J) and MMP‐2 (Fig. 6K, L and M) mRNA expression patterns after MI.

### MMP‐9 colocalized with pPyk2 and LRP1 in the cardiac fibroblasts of ischaemic myocardium at 10 and 21 days after MI

**Figure 5:**
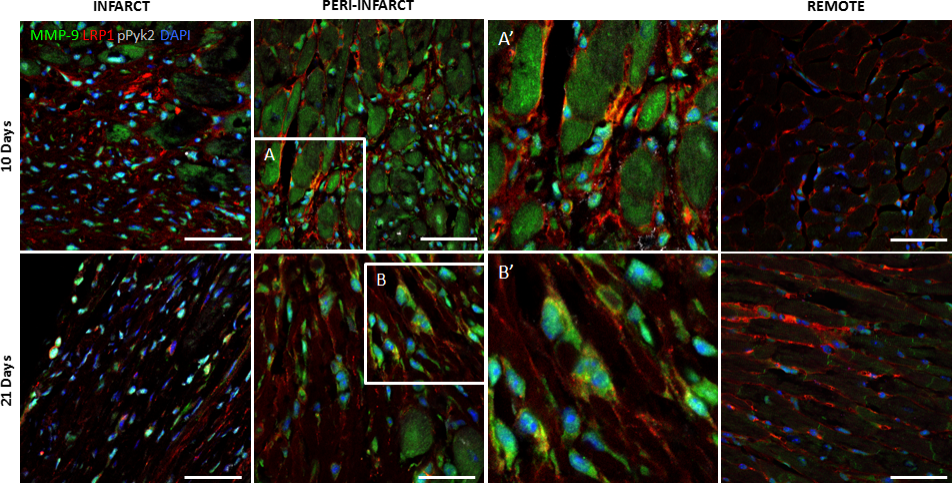
Representative confocal microscopy images showing LRP1, MMP‐9 and pPyk2 levels in the remote, peri‐infarct and infarct zones after MI. Immunostaining of heart cross sections showing the temporal evolution of pPyk2, MMP‐9 and LRP1 expression levels after MI. LRP1 is shown in red; MMP‐9, in green; and pPyk2, in grey. A and B inserts are amplified in A’ and B’, respectively, to show colocalization of the three markers in light yellow. Cell nuclei were counterstained with DAPI (blue). Scale bars, 50 μm.

**Figure 6:**
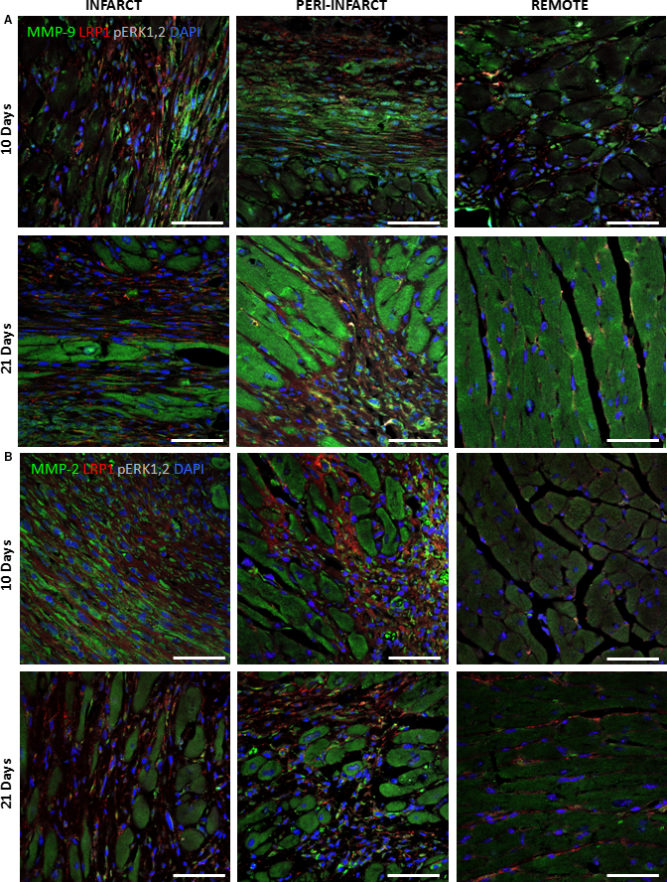
Representative confocal microscopy images showing LRP1, MMP‐9 and pERK1,2 expression levels in the remote, peri‐infarct and infarct zones after MI. (**A**) Immunostaining of heart cross sections showing the temporal evolution of pERK1,2 (grey), MMP‐9 (green) and LRP1 (red) expression levels after MI. (**B**) Immunostaining of heart cross sections showing the temporal evolution of pERK1,2 (grey), MMP‐2 (green) and LRP1 (red) expression levels after MI. Cell nuclei were counterstained with DAPI (blue). Scale bars, 50 μm.

Confocal microscopy images demonstrated a strong colocalization among pPyk2, MMP‐9 and LRP1 in cardiac fibroblasts in the peri‐infarct and infarct zones at 10 and 21 days after MI (Fig. 5). In contrast, pERK1,2, which exhibited only slight expression, did not colocalize with MMP‐9 or LRP1 in any zones or stages after MI (Fig. 6A). Confocal microscopy also demonstrated no colocalization between MMP‐2 and pERK1,2, and only few cells expressed both LRP1 and MMP‐2 in all the areas analysed (Fig. 6B).

**Figure 7:**
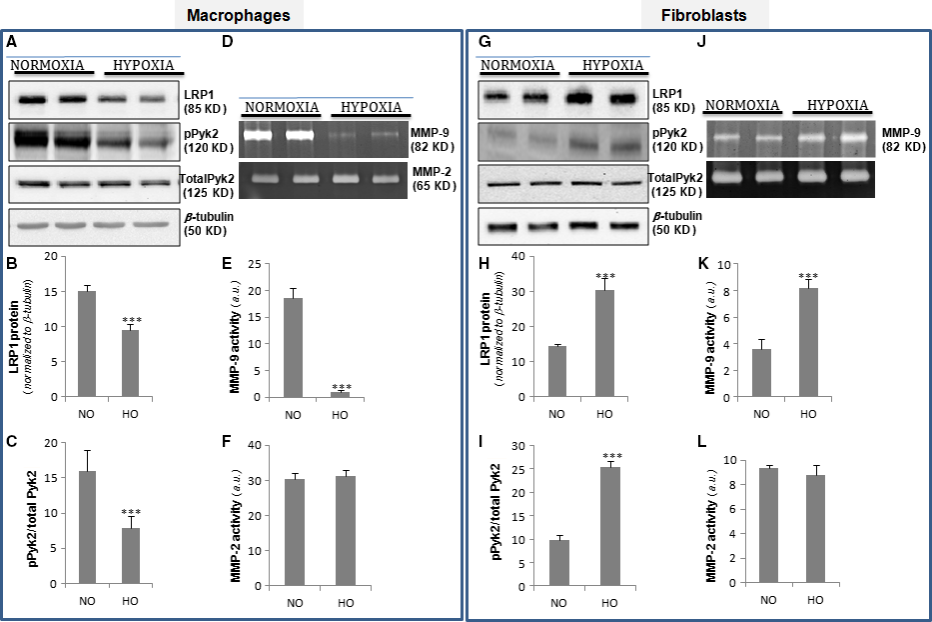
Comparison of the hypoxia impact on LRP1, pPyk2 levels and MMP‐9/MMP‐2 activation in macrophages and fibroblasts. Quiescent macrophages and fibroblasts were exposed to normoxic or hypoxic conditions for 18 hrs. Representative Western blot analysis showing LRP1, pPyk2, total Pyk2 and β‐tubulin bands (**A**&**G**). Bar graphs showing the mean ± S.D. of LRP1 normalized to β‐tubulin levels (**B**&**H**) and pPyk2/total Pyk2 ratio (**C**&**I**). (**D**&**J**) Representative zymography analysis showing MMP‐9 and MMP‐2 activity and bar graphs showing the mean ± S.D. of MMP‐9 (**E**&**K**) and MMP‐2 (**F**&**L**) activity levels. Results are shown as the mean ± S.E.M. of three independent experiments performed in triplicate. ***P < 0.001 *versus* normoxia. NO: normoxia; HO: hypoxia.

**Figure 8:**
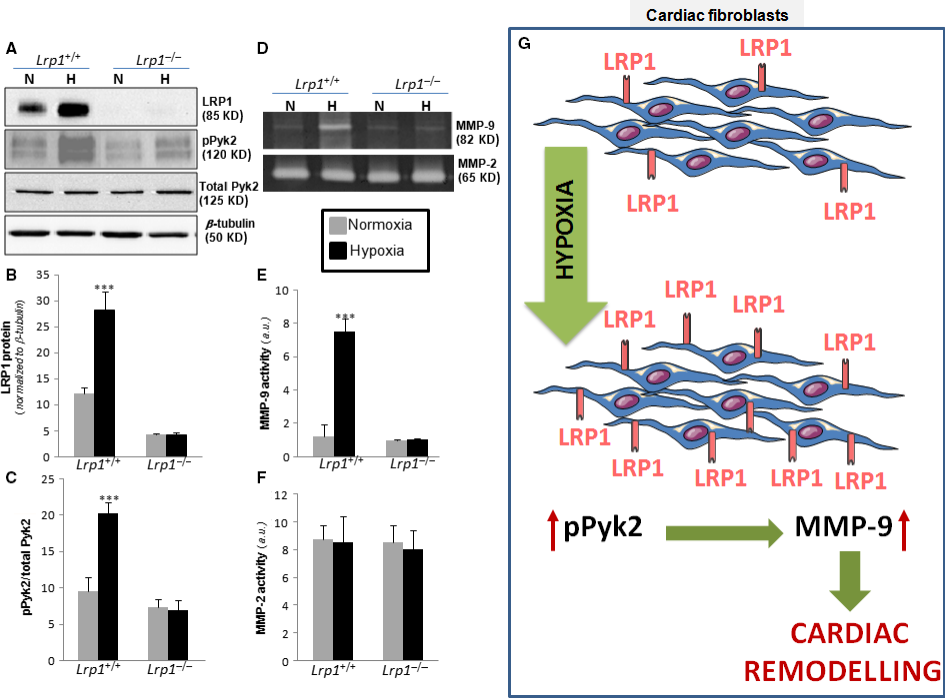
Comparison of the hypoxia effect on pPyk2 levels and MMP‐9/MMP‐2 activation in control (*Lrp1* ^+/+^) and LRP1‐deficient fibroblasts (*Lrp1* ^−/−^). Quiescent *Lrp1* ^+/+^ and *Lrp1* ^−/−^ fibroblasts were exposed to normoxic or hypoxic conditions for 18 hrs. (**A**) Representative Western blot analysis showing LRP1, pPyk2, total Pyk2 and β‐tubulin levels and bar graphs showing the mean ± S.D. of LRP1 levels normalized to β‐tubulin (**B**) and pPyk2/total Pyk2 ratio (**C**). (**D**) Representative zymography analysis showing MMP‐9 and MMP‐2 activity levels and bar graphs showing the mean ± S.D. of MMP‐9 (**E**) and MMP‐2 activity levels (**F**). Results are shown as the mean ± S.E.M. of three independent experiments performed in triplicate. ***P < 0.001 *versus* normoxia. (**G**) Representative scheme showing the crucial role of LRP1 up‐regulation in hypoxic cardiac fibroblasts in cardiac remodelling.

We next compared the role of hypoxia on LRP1 levels in cultured macrophages and fibroblasts. Western blot analysis showed that hypoxia significantly reduced Pyk2 phosphorylation (Fig. 7A and C) concomitantly with a decay on LRP1 protein levels (Fig. 7A and B) in macrophages. In contrast, both LRP1 protein (Fig. 7G and H) and Pyk2 phosphorylation (Fig. 7G and I) levels were significantly up‐regulated by hypoxia in fibroblasts. Zymography analysis showed that hypoxia reduces MMP‐9 activity in macrophages (Fig. 7D and E) but induces MMP‐9 activity in fibroblasts (Fig. 7J and K). MMP‐2 activity levels were similar in both macrophages (Fig. 7D and F) and fibroblasts (Fig. 7J and L). In addition, we compared the hypoxic effects on control (*Lrp1* ^+/+^) and LRP1‐deficient (*Lrp1* ^−/−^) fibroblasts. As shown in Figure 8, hypoxia failed to up‐regulate LRP1 (Fig. 8A and B) and Pyk2 phosphorylation (Fig. 8A and C) in *Lrp1* ^−/−^ compared to *Lrp1* ^+/+^. Moreover, MMP‐9 activity (Fig. 8D and E) was significantly up‐regulated by hypoxia in *Lrp1* ^+/+^ but not in *Lrp1* ^−/−^ fibroblasts, indicating that LRP1 is essential for Pyk2 phosphorylation and MMP‐9 activation in cardiac fibroblasts. MMP‐2 activity levels (Fig. 8D and F) were similar in all tested conditions.

## Discussion

LRP1 has been described as a key regulator of extracellular matrix remodelling 24, 25, 26, 27, 28, which is associated with morbidity and mortality after MI 29, 30, 31; however, no previous studies have analysed the spatial and temporal evolution of LRP1 after MI. Here, we report for the first time that the myocardial LRP1 strongly colocalizes with pPyk2 and MMP‐9 during the fibrotic stages of remodelling after MI. Although it is known that MMP‐9 activity plays a key role in remodelling after MI, these findings have not successfully facilitated improvements in human MI management. Our results indicate that LRP1 is a target for specifically modulating cardiac fibroblast MMP‐9 levels during the early stages of fibrosis after MI.

We demonstrated that LRP1 levels are extremely high in cardiac fibroblasts during the fibrotic states of remodelling after MI. In contrast, LRP1 was scarcely detected in the infarct areas during the inflammatory phase of remodelling after MI, whose main cellular components are neutrophils and macrophages. We have previously shown that inflammatory mediators reduce LRP1 levels in human macrophages through sterol regulatory element‐binding protein (SREBP)‐1 up‐regulation 32. The capacity of inflammatory mediators to up‐regulate SREBP‐1 levels has also been demonstrated in mouse macrophages 33, 34. These results suggest that the inflammatory state characterizing the infarct areas at 1 day after MI may be the cause of the reduced LRP1 levels in neutrophils and macrophages. In addition, macrophages with low LRP1 levels may contribute to the exacerbation of the inflammatory process, as it has been previously shown that macrophage LRP1 deficiency contributes to increased MMP‐9, MCP‐1 and TNF‐α secretion in atherosclerosis 35. Additional studies are required to establish whether the above‐mentioned low inflammatory cell LRP1 levels during the first stage of remodelling after MI are a cause of inflammation, a consequence of inflammation, or both. Consistent with the findings of previous studies 20, 36, 37, we observed high MMP‐9 levels in conjunction with neutrophil and macrophage infiltration during the inflammatory phase of remodelling after MI. Our confocal microscopy images demonstrated a lack of colocalization between MMP‐9 and LRP1 during the inflammatory phase after MI. Taken together, these results suggest that the robust MMP‐9 activation observed at 1 day after MI does not depend on LRP1. Remarkably, myocardial LRP1 was significantly up‐regulated in ischaemic areas during the fibrotic stages of remodelling after MI in conjunction with increases in cardiac fibroblast proliferation. It is well known that cardiac fibroblasts adopt a myofibroblast‐like phenotype in response to specific stimuli and that this acquired phenotype plays a critical role in remodelling after MI in the heart 38, 39. One potent stimulus that triggers the development of this myofibroblast‐like phenotype is hypoxia 40, which is also a potent inducer of LRP1 expression in hVSMCs 11, 12 and cardiomyocytes 13, 41. Therefore, hypoxia may contribute to significant LRP1 overexpression in cardiac fibroblasts located in ischaemic areas after MI. It has been previously reported that LRP1 depletion in fibroblasts inhibits transforming growth factor beta (TGFβ) expression 42, 43. LRP1 overexpression in cardiac fibroblasts may thus promote TGFβ signalling, a crucial inducer of the specialized phenotype that fibroblasts acquire in response to injury 44, 45. Therefore, LRP1 may act as an integrator of TGFβ and hypoxia signalling in cardiac fibroblasts.

Here, we observed increases in MMP‐9 mRNA expression and activation in the infarct areas at all time points after MI, consistent with the findings of previous studies 36, 46, 47, 48. Previous studies conducted by our group showed that the up‐regulatory effects exerted by hypoxia on LRP1 determine MMP‐9 activation and that this mechanism is related to Pyk2 phosphorylation in hVSMCs 12. Using confocal microscopy, we observed high level of colocalization among LRP1, Pyk2 and MMP‐9 in cardiac fibroblasts in the infarct zones at 10 and 21 days after MI. Western blot analysis demonstrated significant Pyk2 phosphorylation up‐regulation in the infarct zone at 10 days after MI, which persisted until 21 days after MI. Consistent with this finding, we observed high MMP‐9 expression and activity in the infarct zone at 10 and 21 days after MI. Factors other than hypoxia, including interleukin‐1α, have been reported to contribute to increases in MMP‐9 expression in cardiac fibroblasts 38, 49, 50, 51. Several factors can modulate Pyk2 and MMP‐9 levels in infarct areas independently of LRP1 and hypoxia.

The *in vitro* results comparing the effects of hypoxia on LRP1 and pPyk2 levels in macrophages and fibroblasts match with *in vivo* studies. On one side, LRP1 and pPyk2 down‐regulation in cultured macrophages exposed to hypoxia fits with down‐regulation of these molecules at early post‐infarct stages (1 day after MI) where the main component is inflammation and macrophages. On the other hand, LRP1 and pPyk2 up‐regulation in cultured fibroblasts fits with the high levels of these molecules at later post‐infarct stages (10 and 21 days after MI) where the main component is fibrosis and fibroblasts. MMP‐9 levels were extremely low in cultured hypoxic macrophages.

The high MMP‐9 activation that we have found in infarcted area at 1 day after MI suggests that inflammatory mediators present *in vivo* overcome the potential down‐regulatory effect of hypoxia on macrophage MMP‐9 levels. The activation of MMP‐9 levels found in infarcted areas at 10 and 21 days after MI is coherent with the up‐regulatory effect of hypoxia on fibroblast MMP‐9 activation. In agreement, our immunofluorescence imaging results show a high colocalization degree between LRP1, pPyk2 and MMP‐9 at 10 and 21 days after MI. Further, we showed that MMP‐9 activation by hypoxia does not take place in LRP1‐deficient fibroblasts. Taken together, our data suggest that by up‐regulating LRP1 in cardiac fibroblasts, hypoxia promotes pPyk2 phosphorylation and MMP‐9 activation in LV remodelling after MI (summarized in Fig. 8G).

We have previously shown that both LRP1 silencing and PP2, a Pyk2 phosphorylation inhibitor, abolished hypoxia‐induced MMP‐9 overexpression and activation in hVSMCs 12. We also showed that in contrast to LRP1 inhibition, PP2 expression did not alter vascular cell pro‐inflammatory phenotypes. PP2‐mediated Pyk2 phosphorylation inhibition has been shown to efficiently reverse fibrosis development in a load‐induced cardiac hypertrophy mouse model 52 and to attenuate fibrosis after MI 53. Here, we showed for the first time the temporal and spatial evolution of pPyk2 levels after MI. Our results suggest that pPyk2 could play a crucial role in up‐regulating MMP‐9 activity in cardiac fibroblasts, especially at 10 days after MI. We previously showed that hypoxia‐induced LRP1‐pPyk2‐NF‐kβ activation is important for MMP‐9 activation, but not MMP‐2 activation 12. The MMP‐9 and MMP‐2 promoter elements are very different 50, 54, suggesting that MMP‐9 and MMP‐2 are modulated by different pathways after MI.

In conclusion, our results suggest that LRP1 plays a major role in MMP‐9 up‐regulation in cardiac fibroblasts after MI and highlight the potential role of LRP1 modulation for treatment of cardiac remodelling.

### Clinical implications

It has been consistently demonstrated that MMP activity modification is a key mechanism underlying remodelling after MI 55, 56. However, these results have not successfully facilitated improvements in human MI management. It has been suggested that temporal and cell‐specific MMP inhibition has therapeutic potential. LRP1 may enable clinicians to modulate cardiac fibroblast MMP‐9 levels during the early stage of fibrosis after MI.

## Conflicts of interest

None.
